# Tissue adhesive, ROS scavenging and injectable PRP-based ‘plasticine’ for promoting cartilage repair

**DOI:** 10.1093/rb/rbad104

**Published:** 2023-11-21

**Authors:** Shiao Li, Dawei Niu, Haowei Fang, Yancheng Chen, Jinyan Li, Kunxi Zhang, Jingbo Yin, Peiliang Fu

**Affiliations:** Department of Orthopedics, Shanghai Changzheng Hospital, Naval Medical University, Shanghai 200003, P.R. China; Department of Orthopedics, Shanghai Changzheng Hospital, Naval Medical University, Shanghai 200003, P.R. China; Department of Polymer Materials, School of Materials Science and Engineering, Shanghai University, Shanghai 200444, P.R. China; Department of Orthopedics, Shanghai Changzheng Hospital, Naval Medical University, Shanghai 200003, P.R. China; Department of Polymer Materials, School of Materials Science and Engineering, Shanghai University, Shanghai 200444, P.R. China; Department of Polymer Materials, School of Materials Science and Engineering, Shanghai University, Shanghai 200444, P.R. China; Department of Polymer Materials, School of Materials Science and Engineering, Shanghai University, Shanghai 200444, P.R. China; Department of Orthopedics, Shanghai Changzheng Hospital, Naval Medical University, Shanghai 200003, P.R. China

**Keywords:** cartilage regeneration, granular hydrogel, gelatin, tannic acid, PRP

## Abstract

Platelet-rich plasma (PRP) that has various growth factors has been used clinically in cartilage repair. However, the short residence time and release time at the injury site limit its therapeutic effect. The present study fabricated a granular hydrogel that was assembled from gelatin microspheres and tannic acid through their abundant hydrogen bonding. Gelatin microspheres with the gelatin concentration of 10 wt% and the diameter distribution of 1–10 μm were used to assemble by tannic acid to form the granular hydrogel, which exhibited elasticity under low shear strain, but flowability under higher shear strain. The viscosity decreased with the increase in shear rate. Meanwhile, the granular hydrogel exhibited self-healing feature during rheology test. Thus, granular hydrogel carrying PRP not only exhibited well-performed injectability but also performed like a ‘plasticine’ that possessed good plasticity. The granular hydrogel showed tissue adhesion ability and reactive oxygen species scavenging ability. Granular hydrogel carrying PRP transplanted to full-thickness articular cartilage defects could integrate well with native cartilage, resulting in newly formed cartilage articular fully filled in defects and well-integrated with the native cartilage and subchondral bone. The unique features of the present granular hydrogel, including injectability, plasticity, porous structure, tissue adhesion and reactive oxygen species scavenging provided an ideal PRP carrier toward cartilage tissue engineering.

## Graphical Abstract

**Graphical Abstract:**
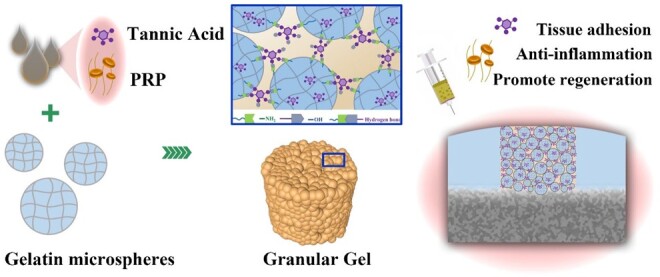

## Introduction

As a common orthopedic condition, cartilage injury usually leads to cartilage resorption, bone destruction, and eventually the development of osteoarthritis [1]. The development of osteoarthritis is accompanied by a large accumulation of reactive oxygen species (ROS), causing further chondrocyte apoptosis, cartilage matrix degradation and deterioration of joint cartilage damage [2]. Cartilage has no blood and lymphatic supply, as well as the low reserve cell density and low cytokines content surrounding the defect, possessing limited potential for self-regeneration [3, 4]. Most patients will eventually experience degenerative changes related to cartilage damage [1]. Commonly used methods of repairing cartilage in clinical practice today include bone marrow stimulation such as microfracture and subchondral drilling. However, the neo-cartilage is abnormal fibrocartilage and has poor biomechanical properties, therefore can only be used as a treatment for symptom relief. Although autologous cartilage grafts have been proposed for decades, donor site limitations and the creation of dead spaces between the columnar grafts during cartilage regeneration have limited their application. Antigenic nature of allografts and the presence of potential disease transmission risks make them difficult to achieve clinical application [4, 5]. Therefore, tissue engineering has been developed for in situ regeneration to treat articular cartilage defect repair. Through the design of tissue engineering scaffold materials, it is not only possible to achieve cartilage damage repair, but also to alleviate osteoarthritis by clearing inflammatory microenvironments such as ROS [6].

Platelet-rich plasma (PRP) is a widely utilized platelet-enriched suspension extracted from blood plasma, renowned for its application in various orthopedic and other medical conditions [7, 8]. PRP has been reported to promote cellular proliferation by secreting growth factors including transforming growth factor β (TGF-β), insulin-like growth factor-1, hepatocyte growth factor, etc., and has been applied in tissue engineering to promote bone, cartilage, skin and soft tissue regeneration [7–10]. Of note, PRP exhibits a favorable impact on the proliferation and differentiation of chondrocytes by secreting chemokines, cytokines and growth factors. Additionally, it possesses the ability to mitigate inflammation and promote the healing of cartilage defects. Thus, PRP has been widely applied in orthopedic injury treatment [11]. Thrombin has the ability to cross-link fibrinogen in PRP to form a solid gel, which enhances the convenience of its clinical utilization. Nevertheless, due to its weak mechanical feature, low attachment to tissue and explosive release of growth factors, injectable carriers that can load and release PRP, fill and adhere to surrounding tissues are more advanced to enhance the efficacy of PRP toward cartilage regeneration [9]. Developing carriers that could load PRP and firmly fix in cartilage defects for PRP release is urgently needed for the clinical application of PRP.

**Figure 1.**
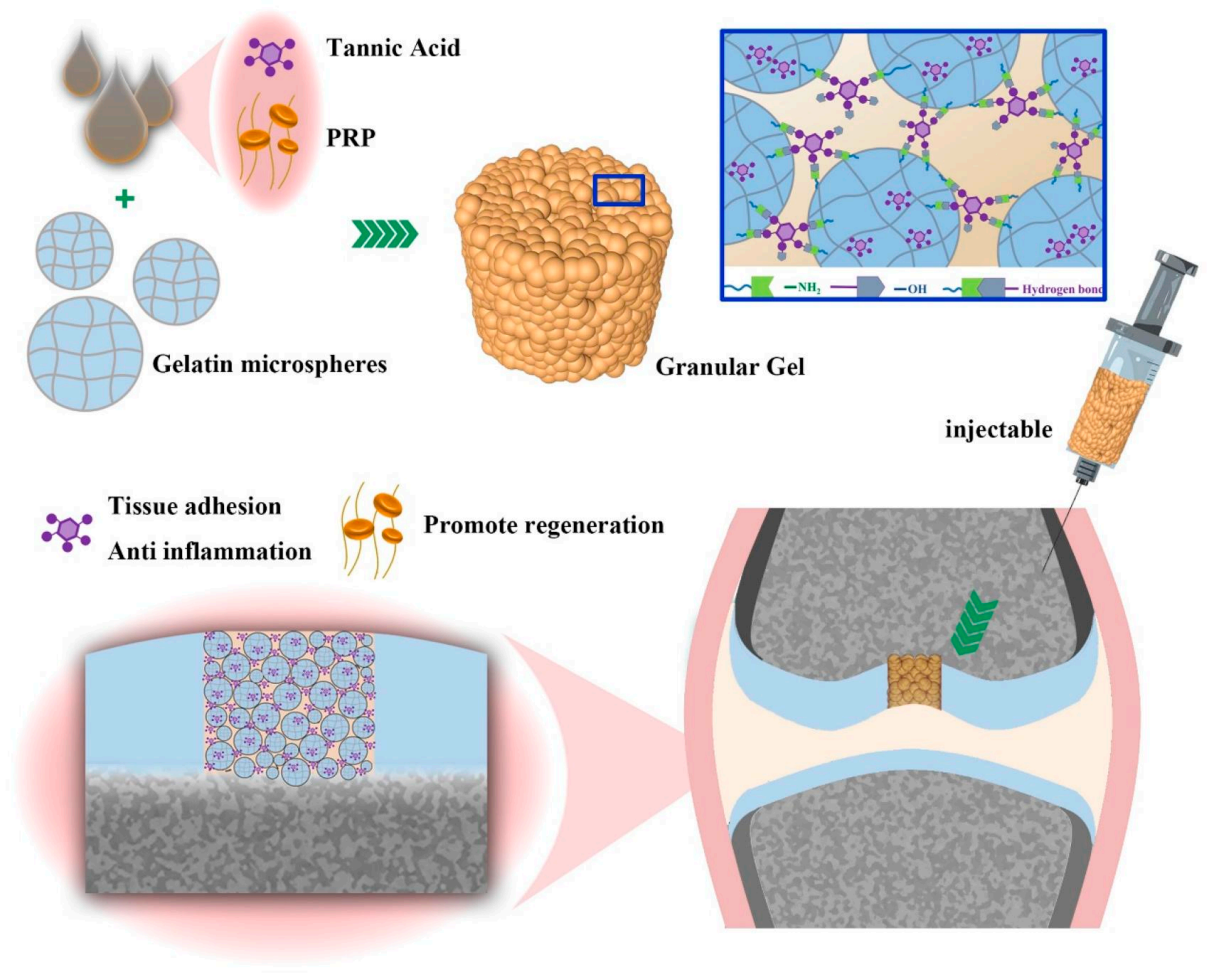
Gel/TA granular hydrogel carrying PRP for cartilage regeneration.

Various hydrogels have been developed for carrying PRP, exhibiting advanced performance such as injectability, stability and continuous release of bioactive component in PRP [12, 13]. Granular hydrogels that aggregate from microgels possess microporous structure, which can promote cell and molecule infiltration. We have developed granular hydrogel based on the mixed chitosan microspheres and alginate microspheres to carry PRP for significantly promoting wound healing [9]. There are two-scale matrix structure in granular hydrogels. One is the nanoscale network matrix within individual microparticles formed from the crosslinking of the intraparticle polymer, which is similar to the bulk hydrogel. The other is the interparticle microporous matrix formed from the aggregated microparticles, which is specific to the interparticle interaction. For one thing, the multi-level structure and porous structure enable the granular hydrogel to rapidly absorb water, which is beneficial to the loading of PRP. For another, reversible covalent actions under mild conditions that are used for the interparticle microporous matrix formation realize the injectability and plasticity [14–16]. However, granular hydrogel assembled from chitosan microspheres and alginate microspheres in our previous study showed no strong tissue adhesion. Thus, it is valuable to develop injectable granular hydrogel with high porosity, which can firmly attach to the damaged part for loading PRP to repair cartilage (Figure 1).

Thus, the present study fabricated gelatin (Gel) microspheres and assembled them by tannic acid (TA) through their abundant hydrogen bonding to fabricate granular hydrogel. Due to the reversible hydrogen bonding, Gel/TA granular hydrogel showed injectability with shear-thinning and self-healing feature. The introduction of TA not only realized the construction of granular hydrogel but also endowed the granular hydrogel tissue adhesion ability. Besides, TA also showed the function of ROS scavenging. The granular hydrogel carrying PRP could be injected into the cartilage defect. Due to the adhesion ability of granular hydrogel, PRP loaded in granular hydrogel could be fixed in defect for release to promote cartilage regeneration.

## Experimental section

### Fabrication and characterization of gelatin microspheres

Gelatin was dissolved in water (2 ml) at 60°C, followed by adding N-hydroxysuccinimide (NHS). Span with the volume of 2 ml and petroleum ether with the volume of 30 ml were mixed under 20 000 r/min for 60 s. Aqueous solution of 1-ethyl-(3-dimethylaminopropyl) carboimide hydrochloride (EDC•HCl) (1 ml) was mixed evenly with gelatin solution and promptly transferred to petroleum ether at a ratio of 1:10 (oil to water) for emulsification for 5 min. Then the emulsion was added to petroleum ether (120 ml) for stirring with the speed of 650 r/s for 12 h. After washing with ethanol and vacuum drying, gelatin microspheres were obtained. Phase-contrast microscope (DM2500, Leica) and scanning electron microscope (SEM) (SU-1500, HITACHI) were used to observe microspheres. The statistics of the microspheres size was carried out by the dimension measurement software during phase-contrast microscope observation.

The dried microspheres were measured (M_0_) and soaked in deionized water for 12 h followed by centrifugation to remove excess water and then weighting of the fully swelled microspheres (M_1_). The ratio of water adsorption (mass ratio) was determined using the equation: WA = (M_1_ − M_0_)/M_0_. The volume swelling was calculated by multiplying the change in volume.

The current study followed a similar fabrication method and used the same component as the microspheres to create bulk hydrogels for mechanical testing. At room temperature, an Instron 5943 testing machine was utilized to conduct a compressive test on cylindrical specimens measuring 8 mm in diameter and 5 mm in height. The applied strain rate during the test was 10% per minute.

### Fabrication of granular hydrogel

Sixty milligrams of gelatin microspheres was placed into a polytetrafluoroethylene mold. TA solutions with different concentrations were dropped into the gelatin microspheres, followed by shaking to make them mix evenly with flat surface. After standing for 10 min, bulk granular hydrogels were obtained. SEM and FTIR were used to characterize the structure and interactions of the granular hydrogel.

Peripheral blood with the volume of 30 ml was taken from New Zealand white rabbits and subjected to centrifugation. The first centrifugation speed was 3500 rpm for 10 min. Upon removing an excess amount of liquid, the residual plasma was meticulously transferred into a tube designed for centrifugation in order to gather PRP. The platelet concentration present in the PRP sample was assessed using a cell analyzer known as BC-2800vet, manufactured by Mindray in China, which was 549 ± 105 × 10^3^/μl. Similarly, for the preparation of Gel/TA-PRP granular hydrogel, PRP (50%, v/v) and TA (10%, w/v) were mixed and dissolved into a solution, which was dropped into the gelatin microspheres followed by shaking to make them mix evenly with flat surface. After standing for 10 min, bulk granular hydrogels were obtained.

### Rheological test

The rheometer fixture was equipped with a flat plate clamp (12 mm) on which granular hydrogel was placed. The relationship between modulus and frequency was examined by scanning frequencies ranging from 0.01 to 100 Hz, utilizing a strain of 0.5%. Scanning measurements of strain were executed at a consistent frequency of 1 Hz, encompassing a strain range of 0.1–500%. The alteration in viscosity was assessed by gradually escalating the shear rate from 0.001 to 300 s^−1^. During alternating strain testing, the G′ and G″ of granular hydrogels were monitored at the alternating strain of 1% and 200% strain. Each strain test was carried out at a frequency of 1 Hz and a scanning time of 200 s.

### TGF-β1 release from the granular hydrogel

Enzyme-linked immunosorbent assay (ELISA) was used to monitor the release of TGF-β1 from granular hydrogel. Briefly, granular hydrogel containing PRP was placed into a tube, followed by the addition of PBS. The PBS was collected every 2 days and replaced with new PBS. The content of TGF-β1 in the collected PBS was quantified by ELISA kits (Westang Bio-tech, Shanghai, China). PRP gel fabricated from the thrombin cross-linked PRP was used as the control.

### Lap shear test

The lap shear tests were carried out to test the adhesion strength of granular hydrogels. Gelatin solution (5 wt%) was spread onto glass slides and dried at 37°C. Granular hydrogels were injected into the overlapped area between two glass slides at room temperature and compressed for 1 min. A tensile machine (DXLL-10000, D&G Measure Instruments, China) was employed to carry out stretching test. The shear velocity was 13 mm/min. The test was stopped when two glass slides were wholly separated.

### Intracellular ROS scavenging test

Human umbilical vein endothelial cells (HUVECs) seeded in a 24-well plate (200 μl/well, 1 × 10^5^ cells) were cultured for 24 h for cell adhesion. Granular hydrogels with the same weight but different TA concentrations (10 wt% and 20 wt%) were placed into the wells of the plate and co-cultured with cells. To conduct the incubation process for 6 h, the wells were treated with Rosup at a concentration of 50 mg/ml. Following this, the cells underwent a washing procedure using a serum-free cell culture medium for three times. Subsequently, the incubation with the Reactive Oxygen Species Assay Kit (ROS Assay Kit, DCFH-DA) was performed for a duration of 20 min. Finally, the observation of the cells was carried out using an inverted fluorescence microscope manufactured by Nikon, specifically the Ti S model.

### *In vitro* biocompatibility test

Rat fibroblasts (purchased from Bluefbio Biology) were cultured using growth medium, Dulbecco’s Modified Eagle’s medium with 10% fetal bovine serum and seeded in the 96-well plates. After 24 h, granular hydrogel pieces with the same weight were placed into wells to co-cultured with cells. At 1, 3, 5 and 7 days, the cells were washed three times with PBS after the removal of granular hydrogel and growth medium. Next, a cell counting kit-8 reagent (CCK-8, Dojindo, Japan) was introduced by adding 10 μl of the reagent into each well. The plate was then incubated for a period of 90 min. Subsequently, the absorbance at 450 nm was measured using a microplate reader (Tecan, Switzerland).

A Live/Dead viability/cytotoxicity kit (Beyotime, China) was used to observe cell viability. After removing the granular hydrogels and culture medium and washing three times with PBS, to perform the plagiarism check, we introduced a staining solution to the wells, comprising 1 μM calcein AM and 1 μM propidium iodide. Following this, incubation of the cells was carried out for 30 min at 37°C in a lightless environment. The red staining denoted dead cells while the green staining denoted live cells. The stained cells were observed using an inverted fluorescence microscope (Carl Zeiss Meditec, Germany).

### Proliferation and differentiation of chondrocytes

To determine the effect of granular hydrogel containing PRP on proliferation of chondrocytes, rat chondrocytes were co-cultured with Gel/TA(10%)-PRP in growth medium for 7 days. chondrocytes were co-cultured with Gel/TA(10%) and chondrocytes without co-culture were used as the control. Cells were collected at 3 and 7 days, followed by full lysis with proteinase K (Sigma) at 56°C overnight. DNA content in the lysate was quantified using the Hoechst 33258 dye (Sigma) on a Microplate reader (SpectraMax M2, Molecular Devices, USA).

Chondrocytes cultured in growth medium, chondrocytes co-cultured with Gel/TA(10%), chondrocytes co-cultured with Gel/TA(10%)-PRP were passaged after trysinization using trypsin (0.05%) and EDTA (0.02%). Chondrocytes at P1 and P4 were collected to detect the expression of aggrecan and COL II genes. Total RNA was extracted from the collected chondrocytes using an RNAprep Micro Kit (TianGen Biotech, Beijing, China), and its concentration was determined by measuring the absorbance at 260 nm. Subsequently, cDNA was synthesized. Real-time polymerase chain reaction (PCR) was performed using a quantitative real-time amplification system (MxPro-Mx3000P; Stratagene, La Jolla, CA) with the SybrGreen PCR MasterMix (Applied Biosystems, Foster City, CA). Gene expression of aggrecan and COL II was evaluated with the following primer sequences: GAPDH 5′-CCCTCAATGACCACTTTGTGAA-3′ and 5′-AGGCCATGTGGACCATGAG-3′; aggrecan 5′-AGGTCGTGGTGAAAGGTGTTG-3′ and 5′-GTAGGTTCTCACGCCAGGGA-3′; COL II 5′-ATGGACATTGGAGGGCCTGA-3′ and 5′-TGTTTGACACGGAGTAGCACCA-3′. Relative expression levels were calculated by normalizing the quantified cDNA transcript level (cycle threshold) to that of the GAPDH. The relative gene expression of chondrocytes at P1 was set as 1.

### *In vivo* cartilage repair

The research committee of Changzheng Hospital granted approval for the experimental protocol (CZEC(2021)-349). At the non-weight-bearing region of the rabbit knee joint, specifically on the femoral patellar sulcus, cartilage defects measuring 4 mm in diameter and 2 mm in depth were intentionally created. The blood and broken tissues around the modeling area were rinsed with physiological saline until there was no active bleeding. One defect was created on one leg. One rabbit carried one defect. Gel/TA granular hydrogel carrying PRP was injected into the defect. The articular capsule, muscle and skin tissue were sutured in turn, the incision was bandaged, the vital signs of the rabbit were observed, and the rabbit was sent back to the cage when it woke up. After surgery and on the first, second and third day after surgery, penicillin was injected into the gluteus maximus muscle to prevent infection. The feeding method was according to the routine feeding method of the animal experimental center. The surgical incision was regularly observed, and the dressing was changed to avoid incision infection. Defects treated with Gel/TA granular hydrogel carrying PRP were named as Gel/TA-PRP group. Defects treated with Gel/TA granular hydrogel without PRP were named as Gel/TA group. Defects without treatment were named as Blank group. Totally 30 rabbits were used. Each group contained 10 animals.

Animals were sacrificed after surgery for 8 and 12 weeks. The surgical site was exposed, and the proximal end of the femoral trochlear joint was cut off as the uppermost edge. The entire distal end of the femur was cut off at the lower edge. Sterile blades were used to remove residual soft tissue around the joint. Three physicians were selected to blindly evaluate the gross specimens according to the International Cartilage Repair Society scoring system, and scores were recorded.

The extracted knee joint was fixed in 4% paraformaldehyde for 36 h, and then subjected to decalcification in 10% EDTA for 4 weeks. After the bone tissue softened, the specimen was washed and placed in ethanol for dehydration for 4 h. Then, the femur portion of the specimen was trimmed in half at the coronal position. The parts with obvious structures of the inner and outer condyles and trochlear groove were selected for paraffin embedding. Finally, continuous paraffin sections were prepared (5 μm) and underwent dewaxing and staining with Hematoxylin & Eosin staining (H&E) and Safranin O-Fast Green staining.

Immunohistochemical staining was performed on other sections: After the sections were dewaxed and hydrated, they were immersed in H_2_O_2_ solution for 15 min. After the treatment with digestive solution for 10 min, it was sealed in 20% normal sheep serum at room temperature for 20 min. Afterward, New Zealand white rabbit COL I, COL II antibodies and corresponding primary antibodies were added and overnight at 4°C. After rewarming at 37°C for 45 min and PBS cleaning, biotinylation goat anti-rabbit IgG (secondary antibody) was mixed and left for 1 h. Then, diaminobenzidine was used to develop the color for 10 min. The degree of color development was observed under the microscope, followed by being washed with PBS, and re-stained with hematoxylin for 2 min. Finally, sections were dehydrated in alcohol and transparent in xylene to complete the sealing process. In order to make the evaluation of cartilage defect repair more accurate and objective, this study used the Modified O'Driscoll Histological score scale for histological scoring.

### Statistical analysis

The mean ± standard deviation was presented for the data, and a *t*-test was conducted for comparison. The GraphPad Prism 9 software (GraphPad Software Inc., San Diego, CA, USA) was utilized for statistical analysis. The experiments were repeated on three occasions. A significance level of *P* < 0.05 was employed to determine statistical significance.

## Results and discussion

### Gelatin microsphere fabrication and characterization

**Figure 2.**
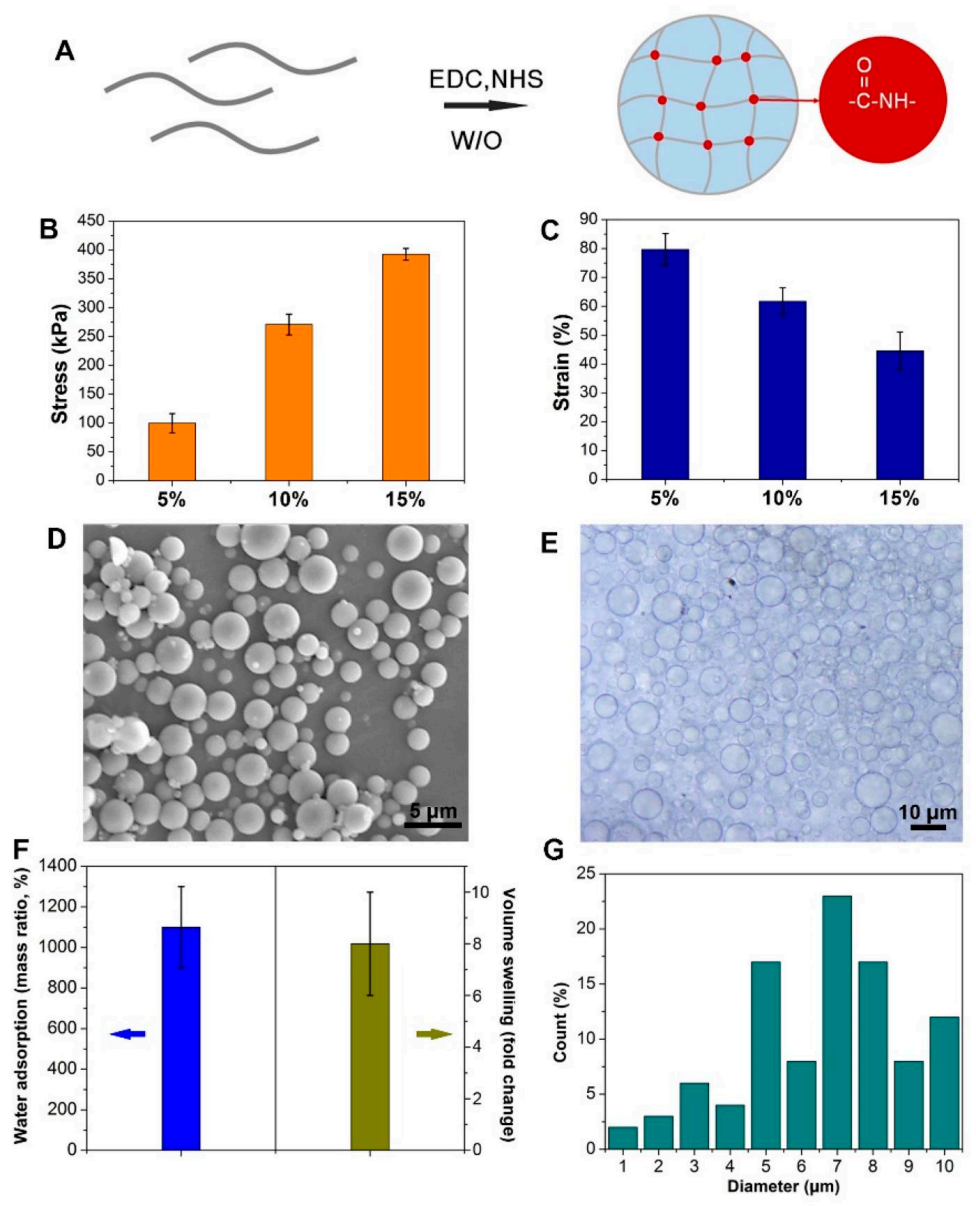
Gelatin microspheres preparation. (**A**) Schematic diagram of gel crosslinking. (**B**) Maximum failure stress of bulk gelatin hydrogels with different gelatin concentration in compression test. (**C**) Maximum failure strain of bulk gelatin hydrogels with different gelatin concentration in compression test. (**D**) The SEM image microsphere (bar scale = 5 μm). (**E**) The wet microspheres observed by phase-contrast microscope (bar scale = 10 μm). (**F**) Swelling behavior of the microspheres. (**G**) The diameter statistics.

Due to the simultaneous presence of carboxyl and amino groups of gelatin, they were cross-linked in aqueous phase with the assistance of EDC-HCl and NHS [17, 18] (Figure 2A). At first, gelatin bulk hydrogels were prepared based on different concentrations of gelatin. Compression mechanics tests confirmed that the higher the concentration of gelatin, the larger the failure stress, but the smaller the failure strain. Therefore, the failure stress of hydrogel with gelatin concentration of 5 wt% was the smallest, while the failure strain of hydrogel with gelatin concentration of 15 wt% was the smallest (Figure 2B and C). Because the granular hydrogel will be subject to shear and extrusion during extrusion, gelatin microspheres are required to have both elasticity and viscosity, as well as good deformation ability. Thus, gelatin with the concentration of 10 wt% was used to fabricate microspheres. Then, gelatin microspheres with the gelatin concentration of 10 wt% were fabricated through the water-in-oil emulsion method. As shown in Figure 2D and E, spherical gelatin microspheres were obtained.

As shown in Figure 2F, the microspheres underwent volume swelling in PBS, with the mass swelling ratio of ∼1100 wt%, while the average volume expanded by ∼8 times. The diameter range of the wet microspheres was 1–10 μm (Figure 2G). Generally, the size distribution area of microspheres prepared by the lotion method can be controlled by the preparation process, such as adjusting the emulsification speed. However, this method can mainly adjust the size of microspheres to a certain range, but cannot achieve complete size unification [14]. This is also the disadvantage of preparing microspheres by the lotion method. The uniformity of size may have an impact on mechanical properties and porous structure [19]. However, the yield of microspheres prepared by the lotion method is higher, which is more convenient for the construction of granular hydrogel.

### Fabrication and characterization of gel/TA granular hydrogel

**Figure 3.**
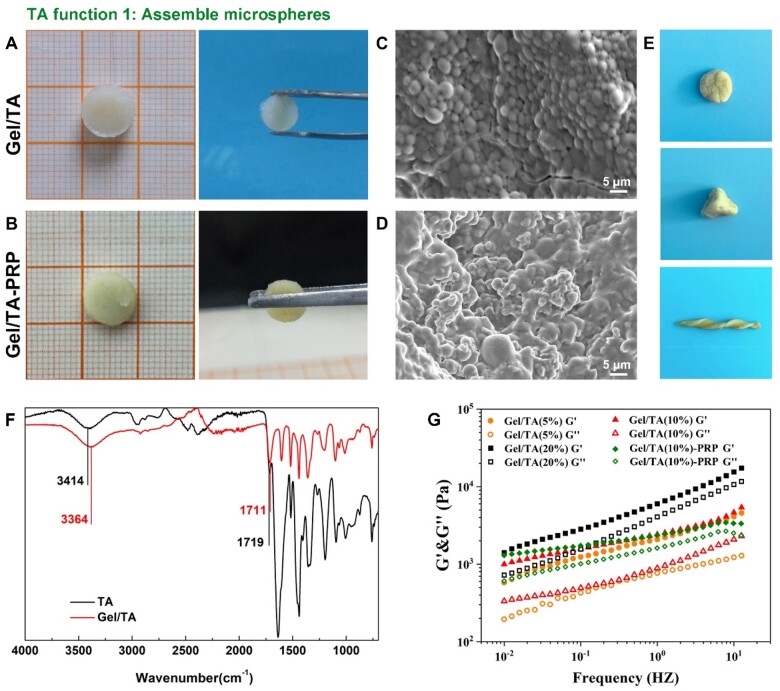
Preparation of gel/TA granular hydrogel. (**A**) General observation of the granular hydrogel. (**B**) General observation of the gel/TA granular hydrogel carrying PRP. (**C**) SEM image of the granular hydrogel (bar scale: 5 μm). (**D**) SEM image of the granular hydrogel carrying PRP (bar scale: 5 μm). (**E**) General observation of the plasticine-like behavior of the granular hydrogel carrying PRP. (**F**) FTIR spectrogram of TA and gel/TA to illustrate their hydrogen bonding. (**G**) The recording of the storage modulus (**G′**) and loss modulus (**G″**) in the scanning range of 0.01–10 Hz (the strain was 0.1%, 20°C).

After the preparation of gelatin microspheres, they were assembled by TA for the construction of granular hydrogel. As shown in Figure 3A, through extensive hydrogen bonding between gelatin and TA [20–22], gelatin microspheres could tightly assemble to form a bulk hydrogel, which was named Gel/TA granular hydrogel. At the same time, the introduction of PRP did not negatively affect the assembly of gelatin microspheres through TA (Figure 3B). SEM observation showed that the microstructure of the bulk hydrogel contained densely packed microspheres (Figure 3C). However, the appearance of PRP made the contour of microspheres less clear (Figure 3D). Of note, the Gel/TA granular hydrogel with PRP behaved like a ‘plasticine’, which could be pinched and maintain various shapes as shown in Figure 3E.

FTIR was used to characterize the formation of hydrogen bonding between gelatin microspheres and TA (Figure 2F). Typical broad peaks presented in the range of 3500 cm^−1^ and 3000 cm^−1^ were the stretching vibration of phenolic hydroxyl (–OH) groups in TA. The absorption peak located near 1711 cm^−1^ was the stretching vibration of the C=O group. The appearance of several peaks at 1602, 1518 and 1440 cm^−1^ was the stretching vibration of C=C. In addition, the characteristic stretching vibration and C–H bending vibration of benzene ring substitution were determined at 1200–1011 and 755 cm^−1^, respectively. In the spectrogram of Gel/TA, the prominent peak at 1719 cm^−1^ belonged to the carbonyl extension of the amide group in the gelatin. The C=O vibration increased from 1711 cm^−1^ in TA to 1719 cm^−1^, indicating that the interaction with hydrogen bonds could affect the enhanced vibration energy of the C=O. The free hydroxyl groups of TA decreased from 3414 to 3364 cm^–1^ due to hydrogen bonding. FTIR showed that there was indeed hydrogen bonding between TA and gelatin, which confirmed that TA assembled gelatin microspheres into the bulk granular hydrogel [23, 24].

Rheological evaluation further showed that the G′ of the granular hydrogels was higher than their G″ in the frequency sweep range of 0.01–10 Hz, revealing that the granular hydrogel was a bulk hydrogel with elastic feature. In addition, the granular hydrogel with a more TA content showed higher value of G′ (Figure 3G). The rheological results showed that the assembly of gelatin microspheres could be realized by TA, resulting in the construction of the elastic solid bulk granular hydrogel. In addition, PRP contains lots of growth factors and proteins. From a chemical perspective, the contents of PRP are similar to gelatin and can undergo hydrogen bonding with TA [23, 24]. Besides, the presence of PRP significantly promoted the solid content of granular hydrogel. Thus, the introduction of PRP improved the G′ of granular hydrogel.

### Rheology properties and injectability of gel/TA granular hydrogel

**Figure 4.**
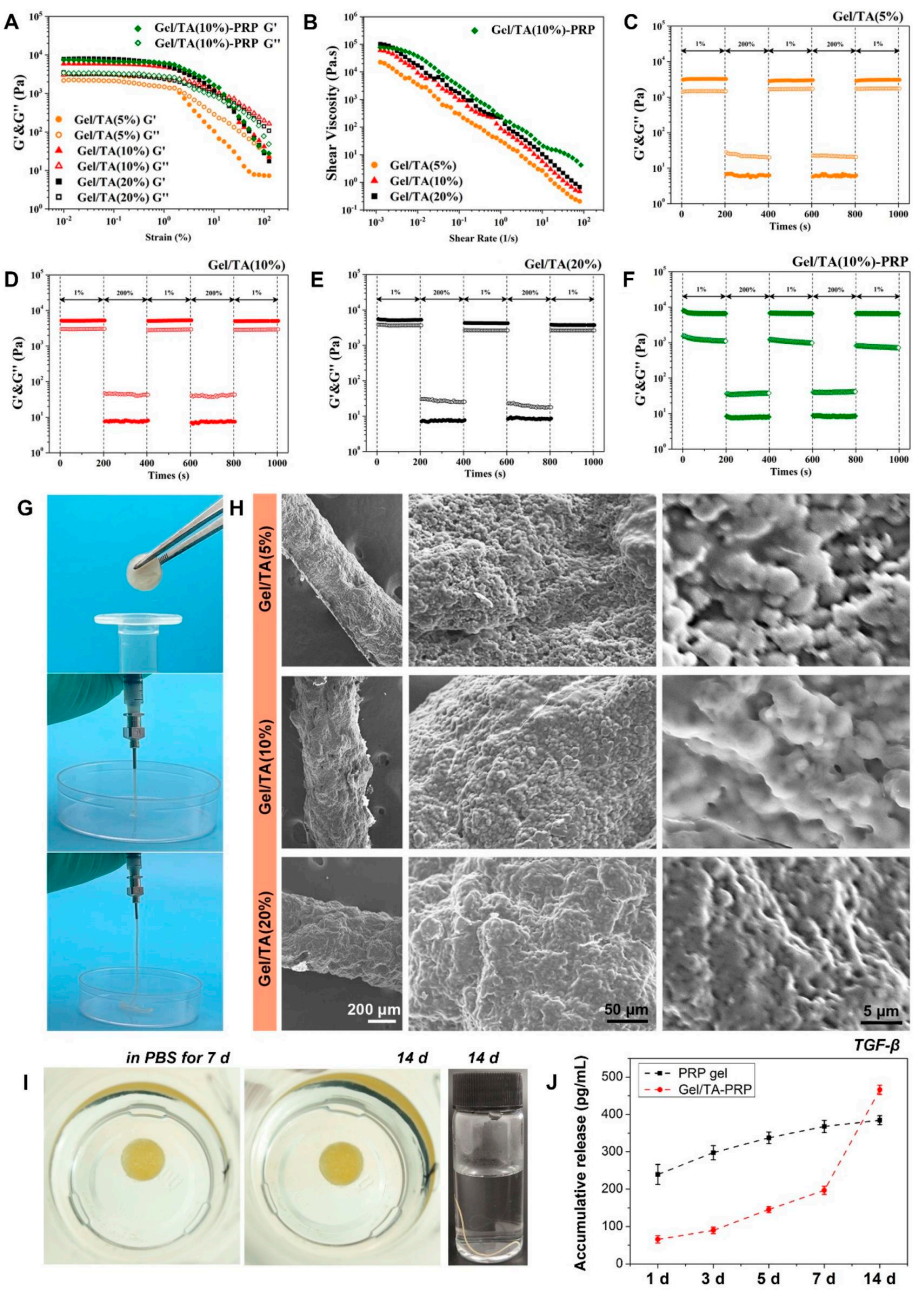
Rheology and injectable properties. (**A**) Oscillatory strain scans of granular hydrogels (0.01–100%, 1 Hz). (**B**) Viscosity changes under shear. (**C–F**) Cyclic strain diagrams of gel/TA and gel/TA-PRP granular hydrogels. (**G**) Extrusion of granular hydrogel with TA concentration of 10 wt%. (**H**) SEM images of extruded filaments with different TA concentration. (**I**) Stability of gel/TA-PRP granular hydrogel immersed in PBS at 7 and 14 days, as well as the stability of extruded gel/TA-PRP filament immersed in PBS for 14 days. (**J**) Accumulative release of TGF-β *in vitro*.

Hydrogen bonding between TA and gelatin not only realized the gelatin microsphere assembly and the formation of bulk hydrogel but also endowed the hydrogel with injectability due to the reversibility of hydrogen bonding. Thus, as shown in Figure 4A, as strain increased, G′ and G″ decreased. G′ decreased more rapidly. The breaking points of the granular hydrogel were determined by the intersection of G′ and G″, indicating the interactions between TA and gelatin microspheres were destroyed, which led to the movement between the internal microspheres. The granular hydrogel changed from a solid state to a ‘fluid’ state. The higher the TA content, the greater the failure strain of granular hydrogel. At the same time, the granular hydrogel showed a shear-thinning feature. Their viscosity decreased with the increase in shear rate (Figure 4B). The higher the content of TA, the greater the viscosity of the granular hydrogel. This shear-thinning feature allowed hydrogel to be easily injected. In addition, the granular hydrogel showed self-healing property. As shown in Figure 4C, granular hydrogel showed G′ higher than G″ under low strain. Under high strain (200%), the granular hydrogel was broken, with the G′ quickly decreased to lower than G″. When the strain returned to 1%, G′ recovered immediately without a significant decrease during the test cycles. Such self-healing characteristic revealed that the granular hydrogel instantly recovered to a solid state after extrusion, which was conducive to immediate curing after injection, so as to achieve accurate filling in the damaged area. Of note, the granular hydrogel, Gel/TA(10%) containing PRP showed similar shear-thinning and self-healing features, revealing that PRP presence did not significantly affect the rheological properties of Gel/TA.

The rheological test results illustrated that the granular hydrogels were extrudable. Thus, the granular hydrogel with the TA concentration of 10 wt% was transferred into a syringe for injection (Figure 4G). The granular hydrogel could be easily extruded through the syringe with the 18 G needle, forming a filament with excellent continuity. After injection, the extruded hydrogel filament immediately recovered to a solid state. According to the SEM observation, the extruded filament was assembled by the packed microspheres (Figure 4H). However, as the TA concentration increased, the microsphere contour in the microstructure became blurred.

In addition, as shown in Figure 4I, either the Gel/TA-PRP granular hydrogel or the hydrogel filament after extrusion showed reliable stability in PBS, revealing the reliable hydrogen bonding between TA and gelatin. Accordingly, one of the deficiencies of PRP gel during application is the explosive release of growth factors. Using granular hydrogel in the present study could significantly address this issue. As shown in Figure 4J, the release of TGF-β from PRP gel was higher than that from the Gel/TA-PRP granular hydrogel during incubation for 7 days, indicating that the explosive release was limited by hydrogel. On day 14, the TGF-β release from the PRP gel significantly decreased after 7 days, and the cumulative release at 14 days was not much higher than that at 7 days. However, the TGF-β in the Gel/TA-PRP granular hydrogel continued to release after 7 days, and the cumulative release still significantly increased after 14 days. This sustained release behavior was related to the binding of hydrogel network to growth factors in PRP. As proteins, these growth factors in granular hydrogel were bound by TA via extensive hydrogen bonding, which effectively delayed their release [23, 24].

### Adhesion and ROS scavenging functions of gel/TA granular hydrogel

As a natural polyphenol, TA can interact with various matrixes via hydrogen bonding, hydrophobic interactions, etc. [20, 21]. It has been widely used for adhesives preparation. At the same time, TA has the function of ROS scavenging [25, 26]. Thus, to verify the tissue adhesion and ROS scavenging function of Gel/TA granular hydrogel, the lap shear test and ROS test were conducted *in vitro*.

**Figure 5.**
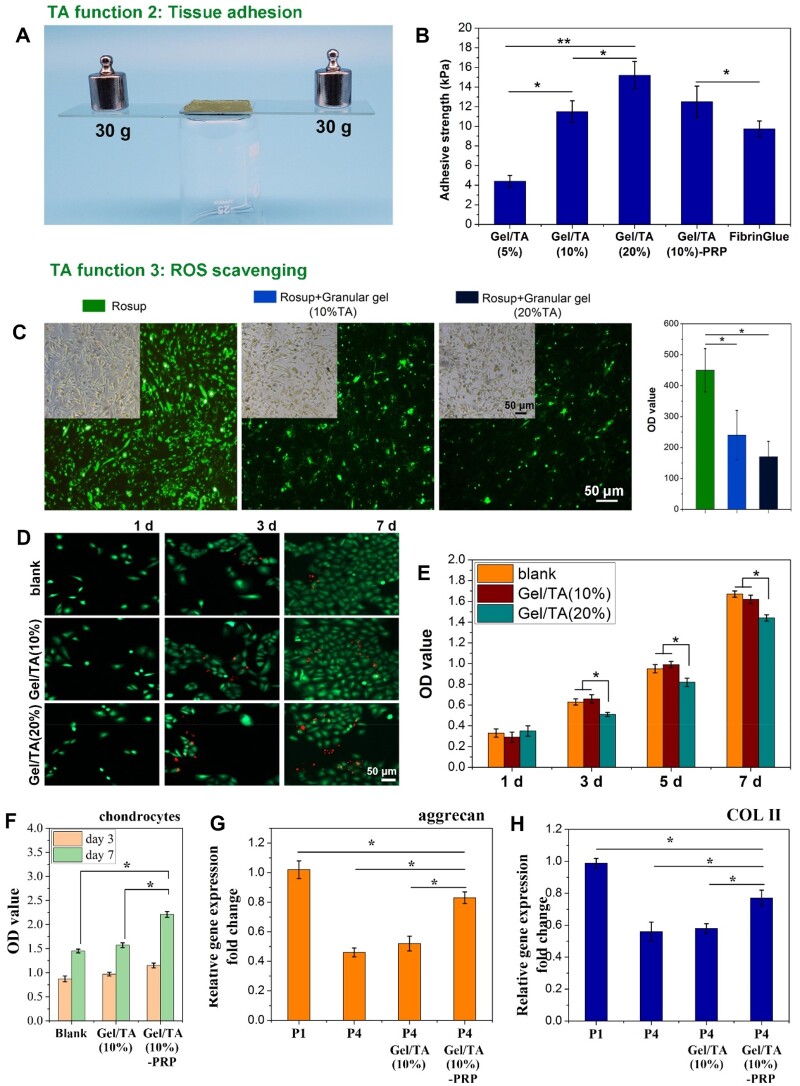
Adhesive strength and ROS scavenging ability of granular hydrogels. (**A**) General observation to show the strong adhesion of the granular hydrogel. (**B**) Adhesive strength of the granular hydrogels and FibrinGlue (**P < 0.01, *P < 0.05). (**C**) Fluorescence pictures and the statistical data of HUVECs cells co-cultured with gel/TA granular hydrogels with different TA concentration, with the presence of reactive oxygen stimulant (rosup), (bar scale: 50 μm, *P < 0.01, *n* = 3). (**D**) Fibroblasts co-cultured with hydrogels subjected to live/dead staining (bar scale = 50 μm). (**E**) OD value to show fibroblasts proliferation *in vitro* (*P < 0.05). (**F**) OD value to show chondrocytes proliferation *in vitro* (*P < 0.05). (**G**) Relative aggrecan gene expression in chondrocytes (*P < 0.05). (**H**) Relative COL II gene expression in chondrocytes (*P < 0.05).

On one hand, Gel/TA granular hydrogel injected onto the glass slide that was coated with gelatin could tightly glue the two glass slides. After gluing, both ends were loaded with 30 g of weights, and the slides were still tightly bonded together (Figure 5A). According to the lap shear test, the Gel/TA granular hydrogel showed higher adhesive strength when TA concentration was higher, revealing the contribution of TA to the adhesion function of granular hydrogel (Figure 5B). Of note, the presence of PRP in granular hydrogel did not significantly affect the adhesion strength. The adhesion strength of Gel/TA(10%), Gel/TA(20%) and Gel/TA(10%)-PRP were all stronger than that of the clinically used FibrinGlue (Porcine Fibrin Sealant Kit, Bioseal Biotech Co., Ltd.). In clinical practice, cartilage grafts usually need to be fixed to fill the cartilage injury site. Therefore, adhesive grafts are more attractive. Therefore, the adhesive PRP graft prepared in this study could better fix PRP to the defect, thereby ensuring the bioactive molecules are released to the wound site.

On the other hand, granular hydrogels (5 mg/ml) were co-cultured with HUVECs (200 μl/well, 1 × 10^5^ cells) that were treated by Rosup (50 mg/ml, 1 μl), which could induce ROS generation in cells, which show positive green fluorescence after staining with ROS-specific probe. The change of ROS content in the co-culture system after the introduction of granular hydrogels can reflect their ROS scavenging function. As shown in Figure 5C, cells treated with Rosup showed strong fluorescence intensity, indicating that there was a large amount of ROS produced in cells. However, the presence of granular hydrogels showed significantly lower fluorescence intensity, confirming the ROS scavenging ability of TA in granular hydrogels.

Usually, cartilage damage often leads to or accompanies joint inflammation. In an inflammatory environment, a large amount of ROS leads to chondrocyte apoptosis, cartilage matrix degradation, and worsening of joint cartilage damage [6]. Therefore, the graft constructed in this study could inhibit ROS while reconstructing cartilage, alleviate the development of arthritis to a certain extent, and create a suitable microenvironment for cartilage regeneration. In addition to the ROS scavenging capacity of Gel/TA granular hydrogels, the presence of PRP might also contribute to the inhibition of ROS through its antioxidant and anti-apoptotic activity [27]. In wound healing study, Farghali et al. found that PRP treatment showed obvious decrease in ROS and redox imbalance with TNF-α and VEGFA genes overexpression [28]. Tognoloni et al. demonstrated that PRP could reduce the oxidative damage of protein and lipid, protecting tenocytes from oxidative stress-induced cell death [29]. Thus, the combination of PRP and Gel/TA would exhibit ROS scavenging ability during cartilage regeneration.

Thus, the *in vitro* evaluation illustrated that the Gel/TA granular hydrogels possessed bulk hydrogel feature, injectability and tissue adhesion ability, as well as ROS scavenging function. Although cells co-cultured with Gel/TA granular hydrogels showed significant proliferation during *in vitro* culture, the Gel/TA granular hydrogel with the TA content of 20% co-cultured with fibroblasts showed more dead cells (Figure 5D). The cellular proliferation was also affected when compared to the blank group and Gel/TA(10%) group (Figure 5E), indicating that the higher content of TA might have a negative effect on cell viability. After comprehensive consideration of the porous microstructure of granular hydrogel, better self-healing performance, higher adhesive strength and better biocompatibility, Gel/TA granular hydrogel with TA concentration of 10 wt% was selected to load PRP for cartilage repair.

### Articular cartilage regeneration *in vivo* by using PRP-loaded gel/TA granular hydrogel

At first, Gel/TA (10%) granular hydrogel carrying PRP was co-cultured with chondrocytes *in vitro*. As shown in Figure 5F, compared with chondrocytes co-cultured with Gel/TA(10%) granular hydrogel and chondrocytes without co-culture, chondrocytes co-cultured with Gel/TA(10%)-PRP showed significantly higher proliferation, revealing its positive effect on chondrocytes proliferation. This was consistent with previous research results that PRP addition to the cell culture medium promoted the chondrocytes proliferation and cartilage matrix secretion [30]. Moreover, cartilage-specific gene expression showed that aggrecan and COL II gene expression levels in chondrocytes significantly reduced after passage, indicating that chondrocytes showed significant dedifferentiation. After PRP intervention, this dedifferentiation was significantly inhibited to a certain extent, confirming that PRP was beneficial for maintaining the phenotype of chondrocytes (Figure 5G and H). PRP is a plasma with enriched levels of platelets, containing high levels of multiple growth factors including TGF-β. The cartilage defect has less cells that can migrate and proliferate. The effect of PRP on chondrocyte proliferation and differentiation might indicate its positive effect on *in vivo* cartilage regeneration.

**Figure 6.**
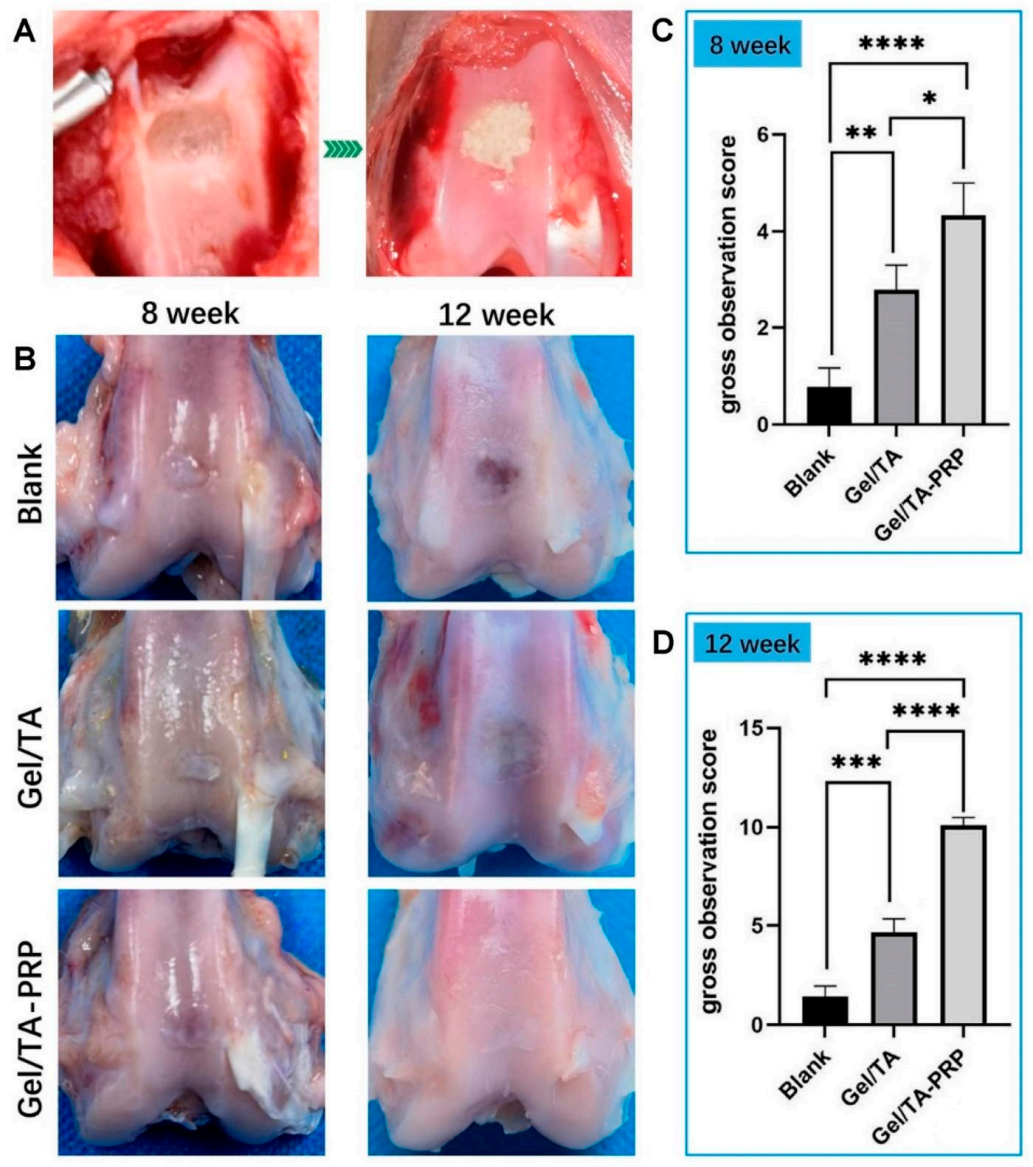
General observation of cartilage regeneration in vivo. (**A**) Articular cartilage defect before and after hydrogel implantation. (**B**) General observation of cartilage repair in three groups at 8 and 12 weeks post-implantation. (**C**) Scoring at 8 weeks. (**D**) Scoring at 12 weeks (****P < 0.0001, ***P < 0.001, **P < 0.01, *P < 0.05).

Then, the Gel/TA granular hydrogels carrying PRP were implanted *in vivo* for articular cartilage regeneration. As shown in Figure 6A, full-thickness cartilage injury without lower bone injury was created and full-filled with Gel/TA granular hydrogels carrying PRP, which was named group ‘Gel/TA-PRP’. Defects treated with Gel/TA granular hydrogels without PRP were used as the control group and named as ‘Gel/TA’. Defects without treatment were employed as the Blank group. One of the important characteristics of granular hydrogels is that they can fill the irregular-shaped damage through injection. Thus, the present study chose the injection of the granular hydrogel to the defect.

At 8 weeks after surgery, a small amount of neo-tissue appeared in the defect area of the Blank group. The boundary with the surrounding healthy cartilage was easy to identify (Figure 6B). The surface of the neo-tissue was uneven. Relatively complete neo-tissues were observed to full-fill in the regeneration area in the Gel/TA group with relatively flat surfaces, forming partial connections with surrounding healthy articular cartilage tissue. But the connection between the regenerative zone and the healthy cartilage plane was slightly uneven. Most of the regeneration areas in the Gel/TA-PRP group had cartilage-like tissue regeneration with relatively flat surface, showing the maximum neo-tissue filling, as well as better integrity and integration with normal cartilage. The general observation scoring results showed that at 8 weeks after surgery, the Gel/TA-PRP group (4.33 ± 0.67) showed obviously higher score than the Blank group (0.78 ± 0.39, *P* < 0.0001) and the Gel/TA group (2.78 ± 0.51, *P* < 0.05). The Gel/TA group showed higher score than the Blank group with statistically significant (*P* < 0.01) (Figure 6C). These results suggested that Gel/TA-PRP had stronger ability to repair cartilage defects *in vivo*.

At 12 weeks post-surgery, there was the smallest area of newly formed tissue in the Blank group, with clear boundaries with surrounding healthy cartilage, uneven surface depressions, and visible layered small cracks in the regenerated tissue. The defects in the Gel/TA group were filled with fibrous connective tissues, with slight depressions visible at the edge of the defect and uneven surface. There was a connection between the neo-tissue and surrounding normal cartilage tissue. The surface of the regenerated tissue in the Gel/TA-PRP group was smooth and flat, with almost all defect areas covered by regenerated tissue with high similarity to the surrounding healthy cartilage in morphology. There were no depressions or cracks in the repaired tissue, showing well-performed integration with the native cartilage tissue, making it difficult to see the boundaries (Figure 6B). The general observation scoring results showed the score of the Gel/TA-PRP group (10.11 ± 0.39) was significantly higher, with statistical significance (*P* < 0.0001), and the significance of the difference increased, confirming a possible positive correlation with surgical time (Figure 6D). The overall score of the Gel/TA group was significantly higher than that of the Blank group (*P* < 0.001). These results suggested Gel/TA-PRP exhibited the strongest ability to repair cartilage defects *in vivo*.

**Figure 7.**
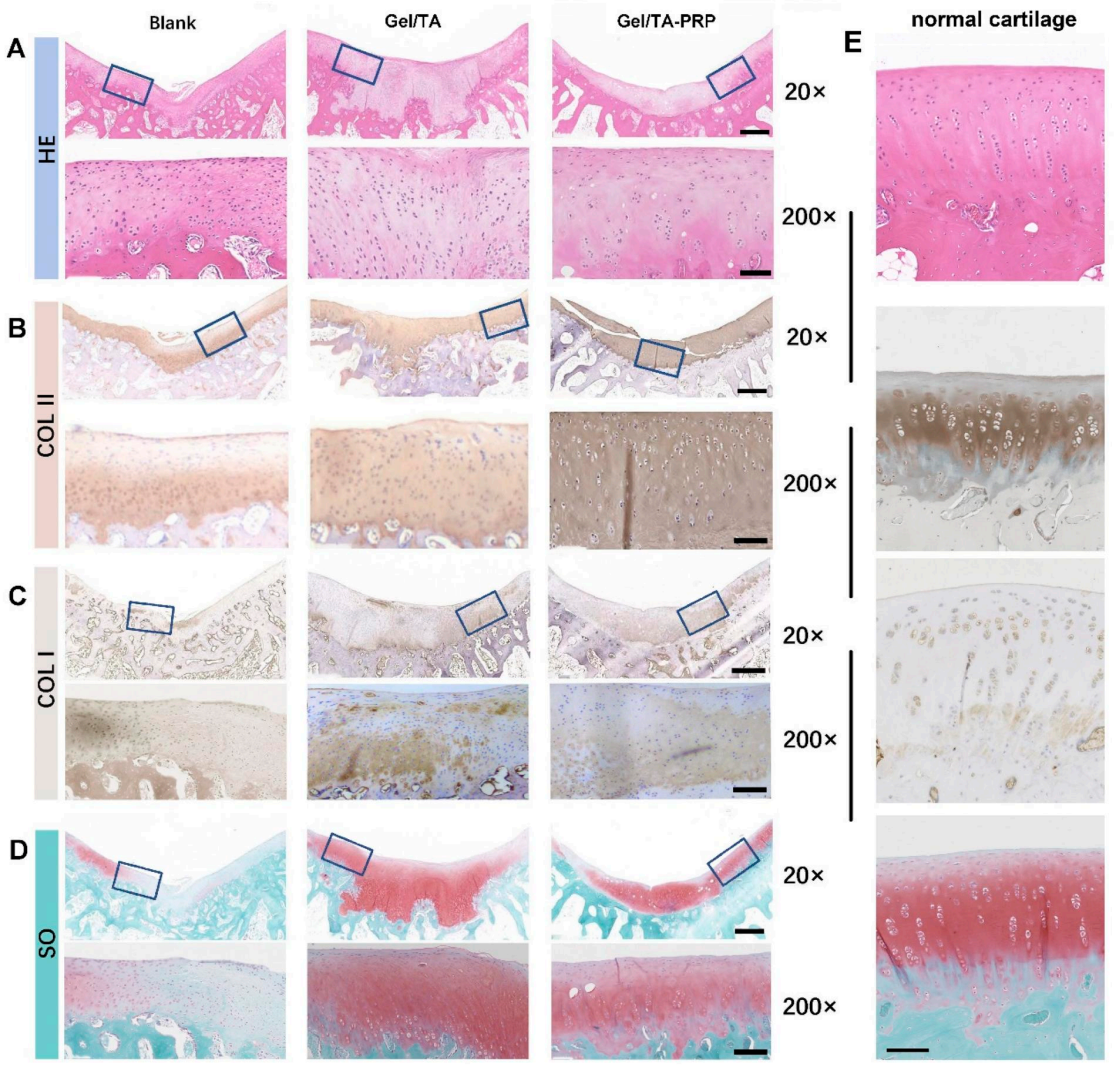
Histological evaluation on samples of different groups at 8 weeks *in vivo*. (**A**) H&E staining. (**B**) COL II immunohistochemistry staining. (**C**) COL I immunohistochemistry staining. (**D**) Safranin O-Fast Green staining. (The scale of a 20× enlarged image was 600 μm; the scale of a 200× enlarged image was 100 μm.) (**E**) H&E staining, COL II immunohistochemistry staining, COL I immunohistochemistry staining, Safranin O-Fast Green staining of the normal cartilage (the scale was 100 μm).

Cartilage regeneration was further evaluated through histological examination. Eight weeks after the operation (Figure 7A), the Blank group was filled with incomplete regenerated tissue in H&E staining, and the connection between cells was loose. The cell morphology was significantly different from that of normal hyaline cartilage cells. The defects in the Gel/TA group were filled with fibrous cells that showed disordered connection with the cartilage matrix. The regenerative cells in the Gel/TA-PRP group were hyaline-like cartilage cells. The thickness of the cartilage was equivalent to the surrounding normal cartilage. COL II immunohistochemistry staining (Figure 7B) showed that the Blank group showed uneven positive staining at the bottom of the neo-tissue, while there was no positive staining in the upper and middle layers. The Gel/TA group showed positive staining at the bottom of the regenerated tissue, but poor staining in the upper and middle layers, with slightly disordered cell structure. The Gel/TA-PRP group showed uniform and scattered positive staining throughout the entire layer, with normal cell morphology. COL I immunohistochemistry staining (Figure 7C) showed that some regenerated tissues in the Blank group were positive, indicating possible fiber regeneration. In the Gel/TA group, light staining was observed in the bottom and upper regenerated tissues, while in the Gel/TA-PRP group, light staining was observed in the bottom regenerated tissues. The results of Safranin O-Fast Green staining (Figure 7D) showed that the Gel/TA-PRP group showed positive and uniform Safranin O-Fast Green staining in the regenerated tissue, while the Blank group showed no staining in the regenerated tissue area. The Gel/TA group showed uniform staining in the middle layer of the regenerated tissue, negative staining in the upper layer and disordered microstructure in the bottom connecting area. The Gel/TA-PRP group was closer to normal cartilage tissue than the other two groups (Figure 7E). The histological score results (Table 1) showed that at 8 weeks post-surgery, the overall score of the Gel/TA-PRP group (14.11 ± 0.69) was higher than the other two groups, with statistical significance (*P* < 0.01). The score of the Gel/TA group was significantly higher than that of the Blank group (*P* < 0.001).

**Table 1.** Histological score for cartilage defect repair (8 weeks)

Group	Score	*P* value
Blank	1.89 ± 0.69	
Gel/TA	7.78 ± 0.96	<0.001
Gel/TA-PRP	14.11 ± 0.69	<0.01

**Figure 8.**
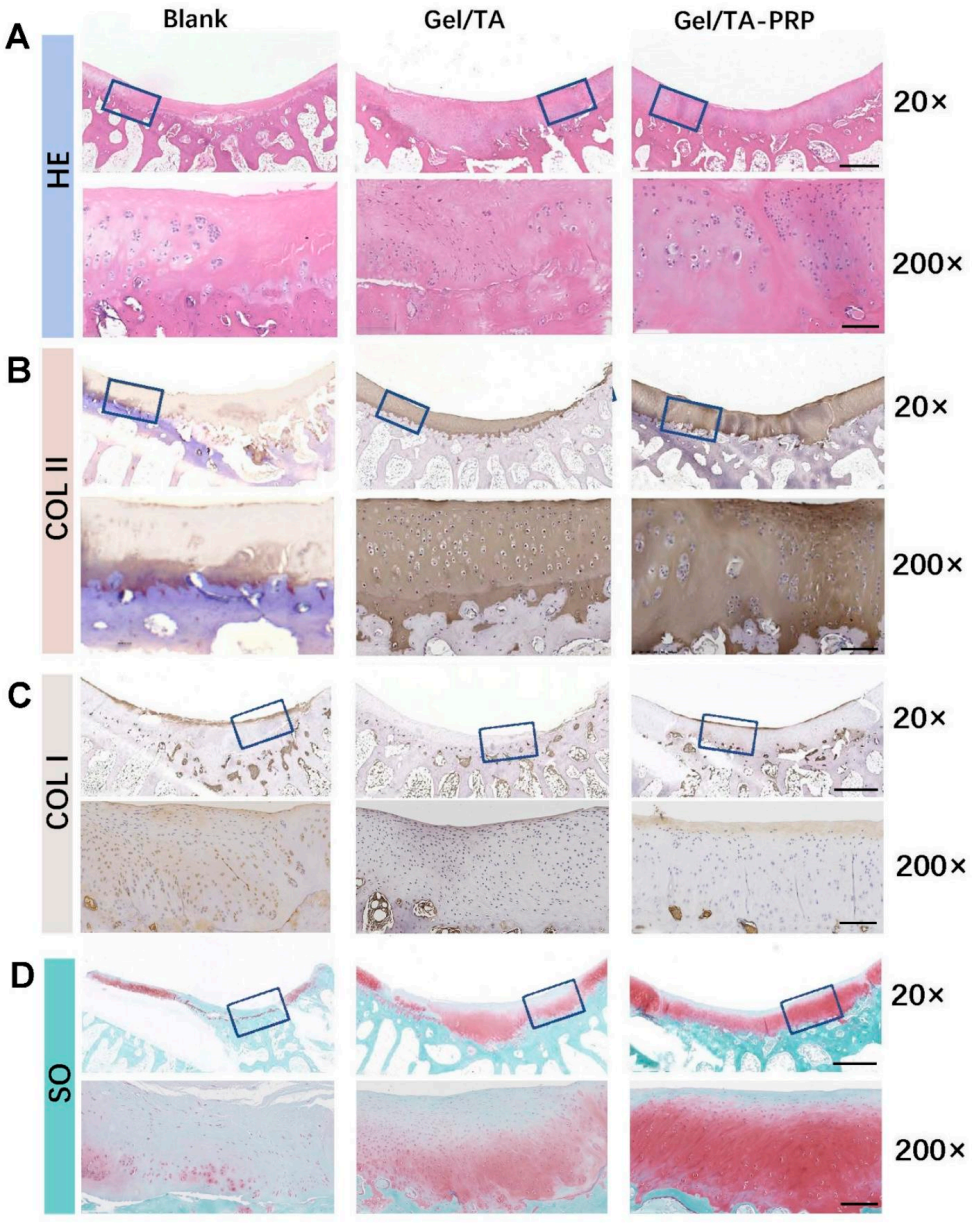
Histological evaluation on samples of different groups at 12 weeks post-implantation. (**A**) H&E staining. (**B**) COL II immunohistochemistry staining. (**C**) COL I immunohistochemistry staining. (**D**) Safranin O-Fast Green staining. (The scale of a 20× enlarged image was 600 μm; the scale of a 200× enlarged image was 100 μm.)

At 12 weeks after surgery, the regenerated tissue in the Blank group was fibrous scar-like and poorly connected to the surrounding healthy cartilage according to H&E staining (Figure 8A). There were a few cracks on the surface of the Gel/TA group, and chondroid-like cells could be observed at the bottom of the regenerated tissue. The thickness of the regeneration in the defect repair area was thicker than that of the surrounding healthy cartilage. The Gel/TA-PRP group had good integration in repairing the defect area, with a smooth surface, and the thickness of the neo-cartilage was basically consistent with the native healthy cartilage. It was well connected to the surrounding cartilage without obvious boundaries. COL II immunohistochemistry staining (Figure 8B) showed that the Blank group had weakly positive staining for uneven regeneration of the entire layer of tissue. The Gel/TA group showed full layer positive staining of regenerated tissue, with lower cartilage thickness and uneven cell distribution. Gel/TA-PRP group showed positive and uniform staining throughout the entire layer, normal cell morphology, and good repair effect. COL I immunohistochemistry staining showed that the upper layer of regenerative tissue in the Blank group was positive (Figure 8C). There was no significant positive staining in the Gel/TA group. The upper part of the regenerative tissue in the Gel/TA-PRP group showed a light positive staining. The results of Safranin O-Fast Green staining (Figure 8D) illustrated that the Gel/TA-PRP group had uniform and strong positive Safranin O-Fast Green staining, indicating that it contained a large amount of proteoglycans. The Blank group only showed positive Safranin O-Fast Green staining on the surface layer, while the Gel/TA group showed a lighter Safranin O-Fast Green staining, indicating a lower content of proteoglycans in it. In the Gel/TA group, by clearing ROS, a more favorable microenvironment was created for cartilage repair. Thus, the neo-tissue was closer to cartilage tissue than the Blank group. However, at 12 weeks, histological examination showed that the cartilage features of the neo-tissue were still insufficient, indicating that without the intervention of PRP, the newly formed tissue lacked cartilage features. Therefore, the Gel/TA-PRP group outperformed the other two groups. Regenerated cartilage tissue gradually grew from the superficial layer in the three groups. However, the growth range in the two control groups was limited. The histological score results (Table 2) showed that at 12 weeks after surgery, the Gel/TA-PRP group’s histological score (20.89 ± 0.509) was higher than the other two groups with statistical difference (*P* < 0.0001). The Gel/TA group showed a statistically significant difference in histological scores compared with the Blank group (*P* < 0.0001). The deficiency of the *in vivo* research lay in the lack of quantitative data on cartilage-specific matrices, which was not conducive to accurately distinguishing the difference between regenerated cartilage and natural hyaline cartilage.

**Table 2.** Histological score for cartilage defect repair (12 weeks)

Group	Score	*P* value
Blank	3.78 ± 0.39	
Gel/TA	12.22 ± 0.69	<0.0001
Gel/TA-PRP	20.89 ± 0.509	<0.0001

In general, the present study used a granular hydrogel based on gelatin microspheres and TA to carry PRP for repairing articular cartilage defects. The present tissue engineering composite exhibited advanced comprehensive performance. For one thing, as an injectable hydrogel, this granular hydrogel was able to more accurately fill in irregularly shaped damages [31, 32]. The more advanced part of the present granular hydrogel was the pore structure among microspheres, which was beneficial to tissue growth and information transmission [14, 16]. At the same time, this granular hydrogel possessed tissue adhesion ability, which contributed to the reliable and stable fixing of PRP in the cartilage defect area. For another, cells such as mesenchymal stem cells and chondrocytes, growth factors (TGF-β, etc.) and drugs (KGN, etc.) were often used for cartilage repair [33–35]. By comparison, the application of PRP in clinical practice is more widespread and has achieved good results. Considering the good clinical application foundation of gelatin, this granular hydrogel showed a guaranteed clinical application prospect as a carrier for auxiliary PRP treatment.

## Conclusion

In summary, the present study prepared gelatin microspheres and assembled them by TA to form a granular hydrogel, which was used to carry PRP for articular cartilage repair. Through extensive hydrogen bonding between gelatin and TA, gelatin microspheres could tightly interact to form a bulk hydrogel, which exhibited extrudability, shear-thinning and self-healing performances. TA not only realized the construction of granular hydrogel but also endowed the hydrogel with tissue adhesion and ROS scavenging ability. The granular hydrogel carrying PRP could be injected into the cartilage defect, exhibiting well-performed plasticity, significantly enhancing cartilage regeneration by the PRP release.
